# MobiPag: Integrated Mobile Payment, Ticketing and Couponing Solution Based on NFC ^†^

**DOI:** 10.3390/s140813389

**Published:** 2014-07-24

**Authors:** Helena Rodrigues, Rui José, André Coelho, Ana Melro, Marta Campos Ferreira, João Falcão e Cunha, Miguel Pimenta Monteiro, Carlos Ribeiro

**Affiliations:** 1 Centro Algoritmi, Universidade do Minho, Campus de Azurém, 4800-058 Guimarães, Portugal; E-Mails: rui@dsi.uminho.pt (R.J.); andresilvacoelho@gmail.com (A.C.);alrmelro@gmail.com (A.M.); 2 Faculdade de Engenharia, Universidade do Porto, Rua Dr. Roberto Frias, 4200-465 Porto, Portugal; E-Mails: mferreira@fe.up.pt (M.C.F.); jfcunha@fe.up.pt (J.F.C.); apm@fe.up.pt (M.P.M.); 3 INESC-ID, Instituto Superior Técnico, Universidade de Lisboa, Av. Prof. Doutor Aníbal Cavaco Silva, 2744-016 Porto Salvo, Portugal; E-Mail: carlos.ribeiro@tecnico.ulisboa.pt

**Keywords:** mobile payments, NFC, user experience

## Abstract

Mobile payments still remain essentially an emerging technology, seeking to fill the gap between the envisioned potential and widespread usage. In this paper, we present an integrated mobile service solution based on the near field communication (NFC) protocol that was developed under a research project called MobiPag. The most distinctive characteristic of Mobipag is its open architectural model that allows multiple partners to become part of the payment value-chain and create solutions that complement payments in many unexpected ways. We describe the Mobipag architecture and how it has been used to support a mobile payment trial. We identify a set of design lessons resulting from usage experiences associated with real-world payment situations with NFC-enabled mobile phones. Based on results from this trial, we identify a number of challenges and guidelines that may help to shape future versions of NFC-based payment systems. In particular, we highlight key challenges for the initial phases of payment deployments, where it is essential to focus on scenarios that can be identified as more feasible for early adoption. We also have identified a fundamental trade-off between the flexibility supported by the Mobipag solution and the respective implications for the payment process, particularly on the users' mental model.

## 1. Introduction

Mobile payments, commonly defined as the use of a mobile device to initiate, authorize and confirm an exchange of financial value in return for goods and services [1], have become a major focus for commercial and research activities in recent years. A large number of trials have been made across many countries, covering a broad range of payment situations, technical approaches and business models. While it is already possible to find multiple payment solutions in many countries, mobile payments still remain essentially an emerging technology, seeking to fill the gap between the envisioned potential and widespread usage. The key reasons for this somewhat limited success seem to be associated with the lack of business models that manage to appropriately combine the many stakeholders involved and the existence of adoption barriers that result from a number of key concerns that people express when faced with the prospect of using mobile payment technologies [2–4]. These adoption barriers go far beyond the mere properties of any particular payment technology, touching issues, such as perceived security, trust and universal access [5].

In this paper, we present an integrated mobile service solution (payment, ticketing and couponing) based on NFC that was developed under a research project called MobiPag. Given our focus, and for the purpose of this particular study, we will define mobile payment as a payment transaction performed through the use of a mobile device using NFC technology. Alternative mobile payment forms are based on Short Messaging Service (SMS) or mobile Internet in which the outcome of the transaction is communicated to the consumer as a text or Internet message [6–8]. This payment method has been used intensively for mobile content, such as ring tones, games, logos and online information retrieval, in which the retailer is remote to the mobile phone user. MobiPag is not specifically conceived for NFC, and most of its infrastructure could be used to support other technologies through the addition of new specific modules. However, in this work, we clearly assume the use of NFC technology as central to the payment process.

The MobiPag Project was conducted by a large consortium of Universities, technology companies, mobile operators, banks and other financial institutions. The most distinctive characteristic of Mobipag is its open architectural model that allows multiple partners to become part of the payment value-chain and create integrated solutions that complement payments in many new and unexpected ways. This openness is an important step towards payment solutions that support flexible business models. In particular, it does not impose any particular collaboration pattern on many different stakeholders that are normally involved in payment solutions, allowing them to adjust their role according to the specificities of particular markets. It also enables many value-added services to be created by combing services from third-party providers into the payment process. An existing Application Programming Interface (API) enables this integration and also leads to the possibility of creating many different applications that provide very diverse views of payment transactions.

The Mobipag system was deployed in a controlled real-world environment at the University of Minho Campus, for a period of one month. The prototype was carefully designed to be a compromise between the simplification needed to run a technology that is still being prototyped and the need to create an evaluation environment that was realistic enough to provide a valuable assessment of the respective user experience. The evaluation has focused on the technical feasibility of the system, including the connection to quality accounts at the participating bank and also on the identification of multiple adoption barriers associated with the realistic user experience of mobile payments. To support usage studies, we designed six mobile payments scenarios corresponding to common day-to-day transactions. These scenarios considered transactions with a broad range of properties, including simple payments, tickets and discount coupons.

The contribution of this work is twofold: (1) we describe the MobiPag open architecture and how it has been used to support a mobile payment trial; and (2) we identify a set of design lessons resulting from usage experiences associated with real-world payment situations with NFC-enabled mobile phones.

Based on the results from this trial, we identify a number of challenges and guidelines that may help to shape future versions of NFC-based payment systems. In particular, we highlight key challenges for the initial phases of NFC-based payment deployments, where it is essential to focus on scenarios that can be identified as more feasible for early adoption. We have also identified a fundamental trade-off between the flexibility supported by the MobiPag solution and the respective implications for the payment process, particularly on the users' mental model.

## 2. Related Work

Existing work on mobile payment systems has largely focused on uncovering the value-chains and qualities that can foster the adoption of mobile payment systems [2–4,9,10]. Kindberg *et al.* investigated users' trust and security concerns in mobile payments using a “electronic wallet” [2]. Ease of use, convenience or social issues were reported as being equally important as trust and security issues. Kristoffersen *et al.* investigated users' attitudes towards m-payments in Norway [3]. The authors concluded that users do not seem to particularly value the mobile payment functionality, except when it enables an immediate use of the product. Mallat *et al.* conducted a study of mobile ticketing service adoption in public transportation [4]. The analysis of the survey suggests that contextual and mobile service-specific features, such as budget constraints, existing alternatives and time pressure, are important deterrents of adoption.

Significant research has been conducted on the development of theoretical research models that examine the factors that determine consumer acceptance of mobile payments [6,11–14]. The work by Chen and Nath [11] presents a framework for m-business applications that is designed to give managers a systematic approach to discovering m-business opportunities in their organizations. Complementarily, the work by Chen [6] proposes and validates a theoretical model that can be used to explain consumers' acceptance of mobile payments and help mobile payment providers identify the key factors that will influence the adoption of their products and services. The results suggest that the main factors determining the adoption of mobile payments are related to perceived usefulness, perceived easy of use, perceived risk and compatibility. Thair *et al.* examined consumer acceptance of m-payments using empirical analysis and comparing acceptance to other traditional online payment systems, such as PayPal and credit cards [13]. In this study, the authors referred to the term “m-payment” as the employment of mobile devices to initiate, authorize, activate and/or confirm online payment transactions by consumers. The results showed that the m-payment method had the highest acceptance by consumers, followed by the PayPal payment method, and that the m-payment method had a positive impact on consumer confidence in trust, cost, control and ease of use. The work by Shin [12] consisted of extending the unified theory of acceptance and use of technology (UTAUT) model in order to explain the development of individuals' behavioral intentions toward the use of mobile wallets. The author referred to the term “mobile wallet” as a form of mobile payment that enables users to share content and access services, as well as conduct payments and ticketing transactions using a mobile phone. This study indicates that perceived security has a strong impact on users' intentions, as well as on their usage behavior, and thus, vendors should establish user trust in mobile wallet security by ensuring that their services are conducted in accordance with users' expectations. To analyze the adoption behaviors of m-payment users, Kim *et al.* [14] proposed a mobile payments research model, which consists of two user-centric factors (personal innovativeness and m-payment knowledge) and four m-payment system characteristics (mobility, reachability, compatibility and convenience). An important result of the study is that there exist clear differences between early and late adopters. Consequently, mobile payment service providers need to apply different business models and strategies depending on the user groups and diffusion stages of mobile payment services. Furthermore, continuous enhancement of mobile payment services may contribute in greater usefulness for users.

Previous research has also studied merchants as adopters of payment solutions. The work by Mallat and Tuunainen [10] explores merchants' adoption of mobile payment services and discusses factors that drive and inhibit their adoption. The results suggest that the main adoption drivers are related to the ability to increase sales or reduce payment processing costs. On the other hand, the main barriers to adoption include the complexity of the system, lack of critical mass, lack of standardization and unfavorable revenue sharing modules.

Research on mobile payments has also addressed the strengths and limitations of various technologies with the objective of proposing new technological advancements to improve mobile payment services and remove identified technical limitations. In particular, NFC is presented as a short-range wireless communication technology that allows two-way contactless communication, offering a faster connection between devices and less chance of interference, making it more secure for use in crowded places. When combined with smartphones, it enables several innovative mobile services, such as payment, ticketing, couponing and access control [15]. Zmijewska studies the adoption factors of ease of use, cost, usefulness, trust and mobility to analyze the suitability of available wireless technologies to create mobile payment systems that the user is likely to accept [16]. NFC is described as being the easiest, the most useful and also the technology with best perception of trust. NFC was also considered a good choice for mobile payments in terms of speed, security and usability when compared with traditional mobile payment service concepts, such as interactive voice response, Short Message Service, Wireless Application Protocol and one time password generator [17]. Ondrus and Pigneur present an assessment of NFC for future mobile payment systems and conclude that NFC is expected to become an enabler for mobile services, more specifically for mobile payments [18]. The NFC-based application presented the best performance results, mainly due to NFC requiring less manual intervention than other technologies. Juntunen *et al.* have conducted a study on the use of NFC technology for mobile ticketing services [19]. They have concluded that mobile ticketing services offer a clear value proposition to travelers and could contribute to the growth of the NFC ecosystem. Rehman and Coughlan developed a mobile payment system to purchase goods at grocers [20]. Geven *et al.* [21] tested the interaction of users with NFC-enabled services, such as door access, payment and information consulting, which showed that user experience was negatively affected by functional failures, missing feedback and inconsistent interaction models. Chen and Chang used the unified theory of acceptance and use of technology model (UTAUT) to explore the factors that affect consumer acceptance of mobile phones with built-in NFC capability [22]. The results indicate that users believe that using the system will help them to attain a gain in job performance if they are well familiar with mobile phone capabilities. Therefore, mobile phone designers should devise easy-to-use interfaces to accommodate this need.

Technical security and trust issues have been addressed in several projects, as for example [23–25]. In particular, Pasquet *et al.* [23] describe the results of a mobile payment trial. The trial was an experiment of an NFC-based payment application that fully supports the international Europay, MasterCard and Visa (EMV) standard. The paper's focus is on the security analysis of several stages of NFC mobile phone payments. One of the main conclusions is that it was difficult for a single organization to ensure the knowledge to cover the whole range of attacks. In particular, it can be complex to provide a solution for the management of mobile payment applications that is secure, standardized and compliant with the intellectual property of the services providers.

There is clearly a great amount of research on mobile payment systems, but there is no clear and well-accepted solution for users, merchants, banks, mobile operators and device manufacturers. It is sufficient that one of them finds no advantage in adopting the system, in order for it to compromise the solution's success. At the technological level, there are still some challenges to overcome. There is a clear need to study the relation between the technological architecture and typical usage situations to overcome the initial barriers of mobile payment service adoption [15,26].

## 3. MobiPag Mobile Payments and Services Platform

The MobiPag platform aims at dematerializing payments with the use of personal mobile devices as an automatic payment terminal that will be interoperable between different financial and national mobile communications agents and able to be universally adopted by users, merchants, shopkeepers and service providers. The MobiPag trial associates all national mobile network operators, a Universal Integrated Circuit Card (UICC) card manufacturer and a major financial institution.

In addition to payments, it also integrates all of the needed logic for merchants and service providers to define their loyalty strategies, including the offer, sale, emission and validation of vouchers, tickets and other value-added services associated with the payment operations.

The supporting technologies, besides the use of mobile devices, include an NFC protocol for communication between the user device and the payment terminal (which can be installed on another mobile device) and an associated secure element, observing and guaranteeing the security of all operations.

### 3.1. System Architecture

**Figure 1.**
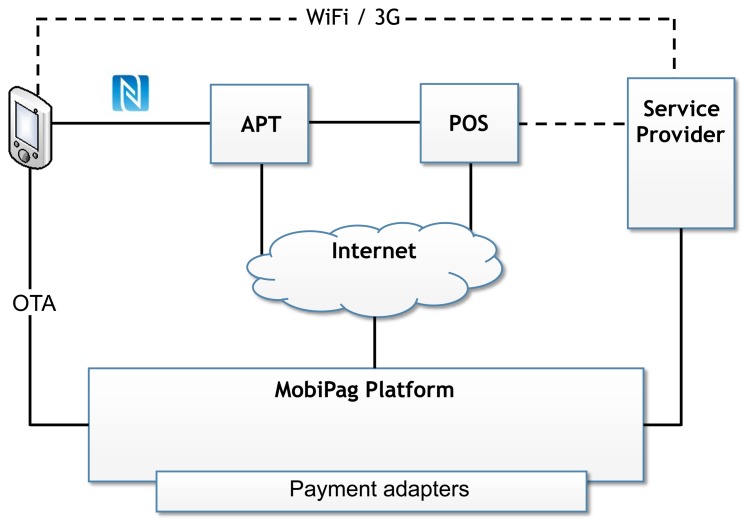
MobiPag architecture: general view.

The system architecture comprises six entities depicted in Figure 1. The customer's mobile device is a phone, a tablet or any other mobile device running the Android operating system, which is capable of communicating via NFC with other devices and contains a secure element, either a secure microSD card (micro secure digital card) or a UICC device, which is able to run java applets in a secure and safe way, inside GlobalPlatform's secure domain [27].

On the merchant's side, the payment infrastructure is usually comprised of an automated payment terminal (APT) and a point of sale (POS) application. The POS application may be integrated in the APT, but its function is well differentiated. The POS interacts with the customer through the APT and with the merchant service provider to agree on the payment amount due in each transaction.

The merchant's APT may be a specific device built for that purpose, similar to current APTs, but with NFC capabilities, or another mobile device, similar to the one carried by the customer, in which case, the transactions between customer and merchant can be framed as person-to-person payments. Similarly, the POS may also be a common merchant's POS or specific POS software running on the merchant's mobile device. This solution targets payments to small merchants, where the actual business logic of the merchant (e.g., stock, price list, *etc.*) can be inside its mobile device.

Financial institutions own financial account services, which are outside of MobiPag's scope, but interconnected with the system. Financial institutions manage the accounts for both merchants and customers, which are identified by virtual cards (vCards), *i.e.*, each customer or merchant possesses one or several of these vCards representing accounts at financial institutions.

Each merchant or group of merchants may deploy additional service providers containing the business logic for other value-added services, such as ticketing or loyalty management, making the architecture fully extensible.

At the heart of the MobiPag payment system is the MobiPag payment backend platform. The backend platform acts as a broker interlinking every service and merchant device, providing facilities to manage all aspects of the associated business model and ensuring compliance with regulation requirements. It contains several payment adapters that connect with each financial account service. Both the merchant's POS and APT can contact the backend platform to make a payment and redeem loyalty and/or service tokens (vouchers, tickets, discounts, *etc.*) provided by the associated external service providers. MobiPag is able to handle several different types of tokens within an opaque construction dubbed vToken.

### 3.2. MobiPag NFC Protocol

All payment and redemption supported scenarios rely on the MobiPag NFC protocol. This protocol is derived from the well-known EMVprotocol [28], simplified by eliminating the negotiating phase. The EMV protocol specifies that the client card is first read and validated by the APT. Then, the payment value is inserted by the merchant and presented to the client, followed by his acceptance and possible PIN (personal identification number) insertion. The payment process terminates when the transaction's success or lack of success and, usually, a printed receipt, are returned. The MobiPag NFC protocol is enriched with a set of features that makes it not just a payment protocol, but a very complete transaction protocol. It is capable of not only assuring the client acceptance of transferring money to the merchant, but also using, redeeming and acquiring other valuables (vTokens) representing vouchers, coupons or tickets. These representations are exchanged between clients and merchants in the same flow (and in addition to money values) of the EMV protocol. This has several advantages from the point-of-view of users and developers. The biggest advantage is the ability to adapt to several transaction use cases, even when they were not initially foreseen. However, it also has some security and development advantages, namely the existence of a single and simple development API and the need to verify the security of a single transaction protocol.

There is a strong security requirement on the APT side. It should be tamper-proof, and it should run only verified and audited software; in essence, it should be the trust anchor of the merchant payment device. Conversely, the trust anchor of the customer is usually some sort of smart card, with essentially the same security requirements, *i.e.*, tamper-proof and running only verified and audited software. The difference between customer and merchant is that there is no POS equivalent on the customer side. Customer interaction is done through the merchant's POS, *i.e.*, it checks the payment value and receives the receipt from the merchant POS. In the MobiPag system, the customers use a mobile device with a display and keyboard of their own and NFC communication capabilities, and the merchant can use a traditional APT equipped with an NFC reader. In the absence of these specialized terminals, the functions of the APT and the POS can be performed by another mobile device.

**Figure 2.**
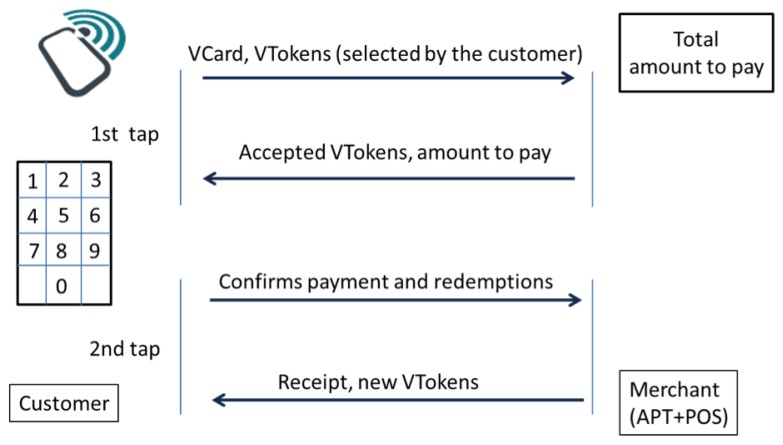
Mobile payments protocol.

This generic messaging protocol between the customer and the merchant may be described in three steps (see Figure 2), separated by two tapping moments, in which both peers explicitly communicate with each other.

1. Preparation: Both users that wish to interact through the payment system (the customer and the merchant) select the virtual cards to use. The customer chooses the virtual card that she wishes to debit and the merchant the set of virtual card types that he is willing to accept. Additionally, the merchant specifies the total amount that he wishes to receive and perhaps some additional information about the transaction;
2. First tap: Within the first tap, two NFC messages are exchanged, one informing the merchant about information set-up by the customer in the previous step and a reciprocal one informing the customer about the information setup by the merchant;
3. Confirmation: The customer takes the information sent by the merchant and confirms the transaction, which may or may not involve the insertion of a PIN code;
4. Second tap: Within the second tap, again, two NFC messages are exchanged; one containing the confirmation from the customer to the merchant and another containing the receipt from the merchant to the customer;
5. Receipt: Finally, both the customer and the merchant are informed of the outcome of the transaction by being shown the receipt of the transaction or an error message.

Embedded in the protocol is the possibility to exchange more information. If the customer possesses some vouchers or tickets that he intends to redeem or validate with this merchant, he can select them on the preparation phase. These will also be transported to the merchant APT and POS on the first tap. The POS can accept them (or ask the associated service provider) and indicate this to the customer, together with the amount to pay (which may be zero, depending on the vouchers/tickets exchanged and the business logic of the service provider).

At the end of the transaction, after confirmation as part of a direct sale or following the loyalty strategy of the merchant, the customer can get new vouchers or tickets granted in this transaction, which will be kept in his device for later use.

It is the backend that intermediates the actual payment and token redemption. The backend receives the request for payment from the merchant. The message should contain the paying customer's vCard and also the receiving merchant's vCard, besides the amount to pay. The vCards identify the owners' account types and financial institution. The message must be signed by both the customer and the merchant, signaling their agreement in this transaction. The backend should now transfer money through the financial institutions' systems.

The request from the merchant can also contain a set of tokens to redeem. These tokens have the service provider identification. It is also the backend's task to contact this service provider in order to redeem the tokens and obtain and send back whatever tokens that are supposed to be given to the customer. The list should contain other tokens previously acquired by the customer and not yet redeemed and, possibly, new tokens acquired in this transaction. In this way, the customer always has an updated list of tokens from the providers. Even if there were a communication or hardware problem in a previous transaction, the customer is always able to recover his account status by performing another transaction (either an acquisition or, for instance, a consultation of a transaction).

### 3.3. Payment Terminal's Architecture

The MobiPag software was designed in a modular and expandable way in order to support multiple payment applications. Besides the interface, these applications, customized for each merchant organization, also incorporate, in the user's point-of-view, the loyalty logic and services. These applications are deployed in the final customer devices and also in the shopkeepers' POSs, providing their users with a personalized experience.

**Figure 3.**
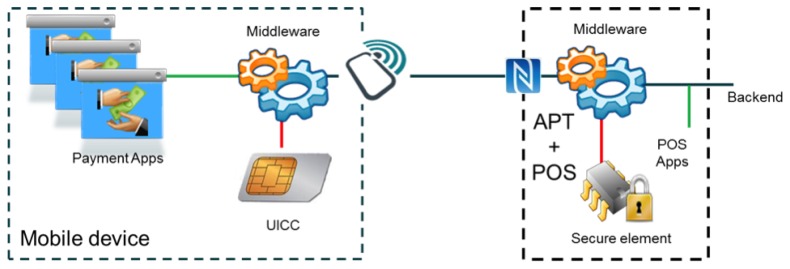
Mobile device and POS architecture.

All of these applications use the same middleware, already present in devices and POSs, exposing the bare minimum of the API needed for starting and performing a payment transaction, including the loyalty elements supported in the protocol. See Figure 3.

The main functionality of the middleware component comprises the function to perform a vCard registration in the secure element, the management of all security related information on the secure element, such as the security codes, secure payments that involve the transfer of money between user and merchant's accounts and token redemption.

The construction, transmission and verification of all messages, and information therein contained, should be done with the guarantee that it could not be appropriated or modified by third parties or repudiated by their emitters. This is done using a tamper-proof secure element, capable of storing sensitive information and performing the appropriate cryptographic operations, ensuring the other needed security requirements.

On the mobile devices, the secure element is either a secure microSD or a UICC. If the APT and POS were also implemented and deployed in a mobile device, the secure element would also be a secure microSD, a UICC or any other device based on smart card technology capable of supporting JavaCard applets and based on the ISO/IEC 7816 and GlobalPlatform standards.

Finally, a MobiPag applet running inside the secure element of both devices offers all of the methods for security-related operations, such as management of vCards and security codes, signing and verification of messages and vToken storage and management.

## 4. The MobiPag Pilot

The MobiPag pilot aimed to demonstrate in a controlled real-world environment the main technologies and services in our mobile payment solution and also to support, from various perspectives, the evaluation of the technology and the respective user experience. The MobiPag pilot was deployed at the University of Minho Campus, in Guimarães, and it was a compromise between the simplification needed to run a technology that is still being prototyped and the need to create an evaluation environment that was realistic enough to provide a valuable assessment of the respective user experience.

### 4.1. The Mobipag Use-Cases

To support the evaluation of the system, we devised six mobile payment situations corresponding to common day-to-day transactions, but designed also to include distinct transaction properties, including simple payments, tickets and discount coupons. These include two scenarios—bus and meal ticket redemption—in which the respective transaction only involves one protocol tap.

All prices, products and services were real, but the users did not pay for them with their own money. Instead, a specific MobiPag account was used and credited with an appropriate initial amount. The threshold for requesting a PIN from users was set at one euro, creating situations with and without a PIN request.

**Figure 4.**
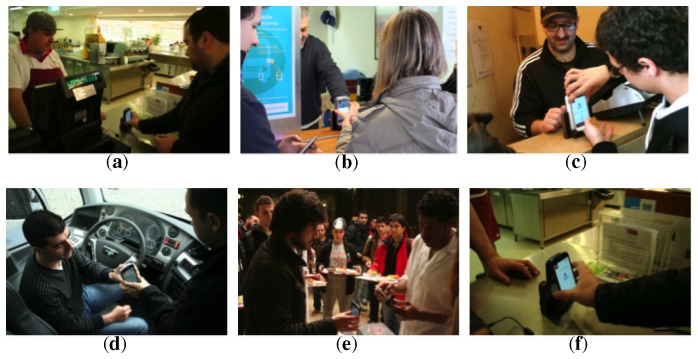
The six payment situations in the MobiPag pilot: (**a**) basic payment; (**b**) buying a bus ticket; (**c**) buying a meal ticket and receiving a coupon; (**d**) bus ticket validation; (**e**) meal ticket validation; (**f**) redeeming a coupon.

We will now describe the interactions performed on each of the six payment scenarios (Figure 4). At the bar (Figure 4a), the merchant registers the requested goods into the merchant's application. The user approaches the merchant's payment terminal with his mobile phone. He receives the payment amount in the mobile phone, confirms and enters the security PIN (if applicable). He approaches the payment terminal with his mobile phone again and receives a payment confirmation message. The transaction is then completed. At the bus ticket counter (Figure 4b), users may buy bus tickets. The transactions are the same, but this time, an electronic ticket is received in the second tap and stored in the mobile phone for subsequent validation in the bus. At the meal ticket counter (Figure 4c), the merchant registers the number of tickets into his terminal. All of the transactions in the previous situations are repeated. The user receives the meal tickets on his mobile phone. In this particular situation, the user also receives a discount coupon. On the bus (Figure 4d), users validate bus tickets stored on the mobile phone. The merchant enters the number of tickets to validate into his terminal. The user selects the corresponding tickets on his mobile phone and approaches the driver's terminal with the mobile device. When the transaction is completed, the user receives a confirmation message. At the meal ticket validation counter (Figure 4e), users pay for their meals with a meal ticket previously acquired and stored on their mobile phone. The merchant enters into his terminal the number of required meal tickets. The user selects the corresponding meal tickets on his mobile phone and approaches the terminal with the mobile device. The transaction is then processed as above. At the bar (Figure 4f), the user selects in his application stored discount coupons before paying. The new total amount is then calculated, and the transaction proceeds as above.

This set of use cases does not exhaust the scenarios in which MobiPag may be used. In fact, several other scenarios may be envisioned and built using the MobiPag API, namely a parent buying bus tickets to be used by his son or the usage of portable ticket inspector terminals, among others.

### 4.2. Technical Approach

In this deployment, the users' and merchants' devices were all Android Samsung Galaxy SIII smart phones with NFC. This particular smartphone model was chosen because it already contained the SIMAlliance Open Mobile API, allowing access to UICC applets. Acting as the secure element, the UICC is capable of storing sensitive information and performing the appropriate cryptographic operations, ensuring the other needed security requirements (see Section 3). Finally, a major bank acted as the financial clearing institution, supplying quality accounts for the merchants and customers, identified by a vCard. The transactions between devices and their financial accounts are brokered and implemented by the MobiPag backend platform (see Section 3).

We have created two mobile payments applications: User MobiPag UMand Merchant MobiPag UM. These applications offer the interface and, from the user's point-of-view, also the logic and services to execute the various payment situations supported in this pilot (see Section 4.1). The merchant MobiPag UM application is actually composed of both the POS and the APT components. The POS interacts with the user and merchant to agree on the payment amount due from the user to the merchant. The APT is the component responsible for communicating with the payment provider through the MobiPag backend platform.

**Figure 5.**
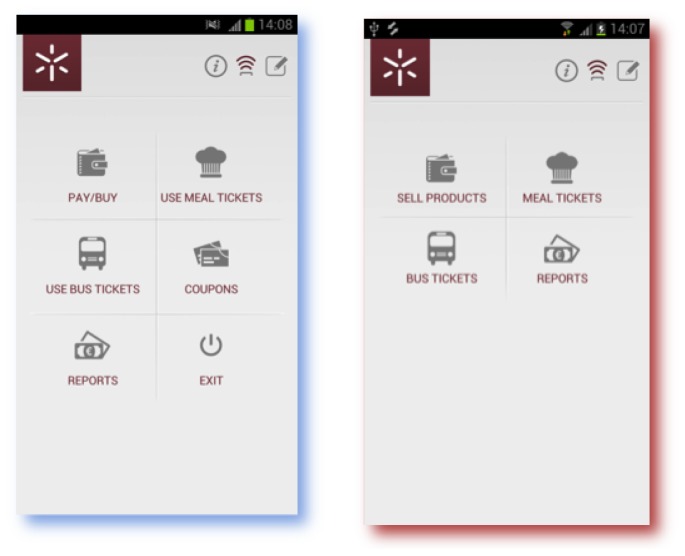
MobiPag main menu application interfaces: (**a**) customer interface; (**b**) merchant interface.

The main menu interfaces of the User MobiPag UM and Merchant MobiPag UM applications are represented in Figure 5. The user app (Figure 5a) allows customers to pay for goods and services selecting the Direct Payment menu. In this menu, they can choose a vCard and the coupons to redeem. It also allows customers to validate meal and bus tickets, by selecting the Meal Tickets and Bus Tickets menus, respectively. Moreover, customers may check the discount coupons that they have stored in their virtual wallet (the Check Coupons menu) and the transactions they have performed (the Reports menu).

The merchant app interface is represented in Figure 5b. The menu, Sell Products, is used in the University cafeterias. The merchant selects the goods he wants to sell (type and quantity), and the application calculates the total price. This option will accept money and discount coupons from customers' devices. Further discount coupons can be sent to customers during the second tap. With the Meal Tickets menu, merchants can sell and validate meal tickets. They only specify which tickets they are going to sell/validate and the quantity. A similar service is performed with the Bus Tickets menu. The Reports menu shows the performed transactions.

## 5. Evaluation Methodology

We ran the overall MobiPag evaluation in two phases. The first phase (laboratory pilot) occurred on 21 March 2013, from 10 a.m. to 4 p.m. This phase involved 9 participants and consisted of performing a set of tasks with the mobile application in a laboratory environment. The main goals of this phase were to test the usability of the application interface and to identify any related problems, particularly with the sequence of taps from the protocol. Considering the results of the first test, the application was improved and the second evaluation phase was initiated.

The second phase (experimental pilot) occurred on 3, 5 and 8 April 2013, from 10 a.m. to 3 p.m. This phase involved 17 customers and 8 merchants. During the experiment, users and merchants were asked to execute all of the payment situations in real-world contexts.

### 5.1. Participants Selection

Participant selection was based on a pre-pilot survey. The main purpose of the pre-pilot survey was to present the MobiPag project, to assess the willingness and viability of the academic community of the University of Minho for integrating the validation scenarios of mobile payments and to recruit volunteers to participate in the pilot. More specifically, the survey aimed at creating an overview of the target population, their age and gender (to realize if it would be possible to have a representative and homogeneous, distributed sample of the population), to understand the sensitivity of the customers in relation to concrete mobile payment scenarios and to gather information on which mobile operators they use. The survey constituted opened and closed questions.

The survey was conducted online (using the Google Docs platform) open to the University of Minho academic community (students, non-teachers employees, teachers and researchers) and was available from 20 September until 16 October 2012. During this period, 317 responses were collected, from which, 112 expressed interest in participating in the pilot. A diverse sample of participants in terms of gender, age and occupation (students, professors and non-teaching employees) was recruited, consisting of 9 people for the laboratory pilot and 17 for the experimental pilot. For the experimental pilot, we have also recruited 8 merchants for the different payment scenarios: Student Support Office, cafeteria, canteen and bus. Merchant selection was done considering the payment situations we wanted to test and their availability to participate.

Fifteen of the participants were aged between 22 and 31 years old, twelve between 32 and 41 years and seven between 42 and 50 years. The majority of them were male (25 in 34), and there were no female merchants. Most of the participants (24 in 34) had high education levels, but the majority of the merchants (5 in 8) only had 12 years of education or less. In the laboratory sample, most of the participants (7 in 9) were non-teaching employees, while in the experimental sample, most of them were students (10 in 17). When asked about smartphone usage, the majority of the participants (25 in 34) answered that they had a smartphone.

### 5.2. Experimental Procedure

The user app and the merchant app were installed on Samsung smartphones and provided to the participants. Both user study phases were comprised of 4 moments:

1. Pilot initial training;
2. Survey for sample characterization with 17 structured questions;
3. User test and observations;
4. Interview with 9 unstructured and 1 structured questions.

#### 5.2.1. Pilot Initial Training

All of the participants in the experimental pilot (customers and merchants) had initial training. The contents of the training were diverse, including a brief explanation of the MobiPag project, of the experiment itself and of the payment and coupon semantics. We have also included a brief explanation of NFC technology and application initiation and configuration. Finally, the different steps to execute the experimental tasks were also described.

#### 5.2.2. Survey

Customers were asked to answer a survey before initiating the experiments. Merchants were also asked to answer the survey during the training day, a week before the experimental pilot. The main goals of the participants' questionnaire were to characterize our sample, to understand how familiar participants were with smartphones and with the University of Minho's social services and buses and what was their opinion about the possibility of using a mobile payments application. The survey had 17 questions. Most of them were closed questions.

The main goals of the merchants' questionnaire were to characterize the sample, to understand how familiar participants were with smartphones and how much interest they had in receiving payments through mobile phone. The survey had 12 questions. Most of them were closed questions.

We collected 34 answers: 9 from participants in the laboratory pilot, 8 from merchants and 17 from customers that participated in the experimental pilot.

#### 5.2.3. User Test and Observations

The main goal of this technique was to directly analyze how the user interacted with the mobile payments system: to understand which payment or validation tasks were more intuitive to execute with the mobile phone; to encounter the main participants' difficulties; and to find out if there were substantial differences between participants. That is, we wanted to draw conclusions about the usability of the application, the users' cognitive competences and how, eventually, a mobile payments application would be accepted by the market.

For evaluating the usability of application and participants' cognitive skills, we have created a list of tasks to be undertaken by participants. At the beginning of the pilot, participants were asked to describe aloud the tasks that they were performing with the application, and at the end, a MobiPag team member took some notes on additional participants' comments. When the participant had any difficulty in externalizing actions, a MobiPag team member would question him/her about the task's execution. Every task was timed, and participants were asked to rate all of the tasks (1, very difficult, to 5, very easy). In some tasks, a MobiPag team member asked the participants about their perception regarding what had happened, when the payment occurred and what had contributed for that perception (application messages, beeps, vibrations or approximation to the POS). During participants' task execution, a MobiPag team element wrote any relevant comments with respect to the manner in which the task was executed.

#### 5.2.4. Interviews

After completing the tasks, at the end of both pilots (laboratory and experimental), customers and merchants were asked to participate in an interview, which was intended, as its central objective, to understand the overall participant perception regarding the use of the mobile payment application, the things participants most liked or perceived as confusing or difficult and suggestions to improve the application. After a brief explanation of NFC technology, we also wanted to understand security perceptions about making payments via NFC. It was also important to get the opinion of the merchants (bar, Student Support Office, canteen and bus). As such, all involved merchants were interviewed. In this situation, we intended to understand if merchants perceived the new technology as an added value to the payment process.

All of the interview excerpts were codified. The codes used for laboratory pilot interviews have “Lab” followed by the participant's code: “Cust + number”. The codes used for the experimental interviews have “Ex” followed by the customer's code “Cust + number” and the merchant's code “Mrch + number”. We will use this notation in the following sections when we are presenting some interview excerpts that are relevant for our analysis.

## 6. Analysis

As stated in Section 1, the purpose of the work presented in this paper is two-fold: (1) to describe the MobiPag open architecture and how the various components can be instantiated into a specific and complete solution to support a mobile payment trial; and (2) to identify a set of design lessons resulting from usage experiences associated with real-world payment situations using NFC-enabled mobile phones. Five relevant topics emerged from the analysis of our empirical results, which we used to structure a set of relevant design lessons: “usability of the payment system”, “perceived value”, “being in control”, “embodiment of the payment system” and “security perception”.

### 6.1. MobiPag Open Architecture Analysis

Although theoretical proof of the security of the MobiPag protocol is beyond the scope of this paper, in this section, we discuss its security claims, security vulnerabilities and countermeasures. We also discuss the MobiPag architecture rational and how it differs from existent mobile payments solutions.

MobiPag proposes a new architecture featuring two layers: a business layer and a payment layer. At the heart of this proposal are two unproven claims. The first claim states that previous proposals have failed, because their benefits, when compared with already deployed payment alternatives, were not enough to overcome the combined costs of every player involved (customers, merchants, mobile network operators, financial institutions). The second claim is that mobile payments combined with mobile application gain an added value that changes the balance between benefits and costs in favor of mobile payments.

In line with the above claims, MobiPag does not feature a mobile payment application; instead, it provides a payment infrastructure that may be integrated with different mobile applications (games, public transportation, merchant loyalty tokens, specific POS applications, *etc.*). Besides the payment itself, these applications take advantage of the ability to exchange securely several different types of “currencies”, encoded into loyalty tokens, public transportation tickets or game points.

Existent mobile payment solutions provided by the main credit card emitters (VISA/payWave, MasterCard/PayPass, American Express/ExpressWay) are payment-only solutions, without including or directly allowing the added value for the customer and merchants, which is supporting in the same gesture, protocol and transaction the acquisition, exchange or redemption of other values, besides money, as MobiPag supports. Google Wallet is evolving in this direction, allowing the association of other functionalities with a stored value account (SVA) or credit card-based payments.

The MobiPag payment layer is comprised by a protocol, run by the mobile phone, and a secure storage system run by a secure element (a UICC). We argue that this protocol and storage architecture are flexible enough to support several types of applications; we have designed a few, implementing different use cases to support this claim.

MobiPag's security anchor is the secure element. Messages exchanged by the protocol are generated and signed within an applet running in the secure element that also stores the cryptographic keys and caches a copy of the tokens owned by the user (for offline redemption). The messages generated in one applet must be validated in the other, rejecting replays, false generations or message tampering, occurring outside the UICCs.

The signed message that issues the payment (the third message of the four exchanged ones; see Figure 2) contains a serial number and the identity of the destination merchant; therefore, it cannot be forged or replayed to the same or to another merchant, even if the attacker takes control of the user's mobile phone.

MobiPag is vulnerable to two types of relay attacks, although it contains mitigation mechanisms against one of them. The relay attack against which MobiPag has no security defense is the less dangerous one and the most common one among payment systems. It happens when a user is persuaded to tap her phone against a false merchant and the false merchant is relaying the communication to a real merchant through another NFC tap; the user perception is that she is paying to one merchant, but she is, in fact, paying to another.

A more dangerous relay attack happens when the attacker is able to relay the payment without requiring the user to tap her phone. For that, the attacker is required to run an application in the user's smartphone (e.g., malware) that communicates directly with the secure element. To prevent this, MobiPag takes advantage of a feature of the GlobalPlatform Secure Element Access Control specification [27], which restricts the applications allowed to access the secure element (*i.e.*, the UICC). However, this feature is effective only if the operating system was not compromised and tampered with, such that it allows running one app with the hash and signature of another. Rooting the Android operating system can allow attacks that ultimately could lead to this kind of tampering. In MobiPag, only the middleware service is allowed to access the applet inside the UICC.

MobiPag uses the NFC peer-to-peer mode to convey messages between the customer and the merchant, instead of the more standardized emulation mode, for two reasons. The first one is that it allows the customer to play the role of the merchant for small payments (person-to-person payments), which is not possible in emulation mode (although this functionality was not implemented in the pilot, nor yet supported by the backend and financial institutions). The second one is that most relay attacks described for ISO 14443-A complying devices [29] are avoided, given that in peer-to-peer mode, the NFC controller never talks directly with the secure element, nor are the payment applications and middleware activated automatically when close to a reader. The MobiPag protocol is not directly compatible with the ISO 14443 protocol.

### 6.2. Overall Usability of the Payment System

Table 1 shows the average time users took to complete each task and the average rating they assigned to tasks. The laboratory pilot's users classified almost all tasks as “easy” or “very easy” to perform, except for Tasks 2 and 10. These tasks were also those that took longer to be completed. Participants successfully completed 86 tasks of 90, but there were four tasks (4.4%) not completed. Two people failed to complete Task 10, and another person failed to complete Tasks 2 and 3.

During the laboratory pilot experiment, some problems concerning application user interfaces were identified, such as misleading menu names or lack of feedback.

Before proceeding with the experimental pilot, we improved the application user interfaces based on users' suggestions and on our own observations. Hence, the names of the menus were changed in order to become more intuitive, and further feedback messages were included. According to Table 1, the average time that users took to perform each task in the experimental pilot decreased dramatically, taking less than a minute to complete. Furthermore, users found all tasks to be “easy” or “very easy” to perform. All 160 tasks (16 users × 10 tasks per user) were completed successfully, which translates to an improvement when compared with the previous phase: the number of times users selected the wrong menu dropped from 22% to 2.5% (four tasks in 160).

The major problems identified in the laboratory pilot phase were related to the performance of the NFC connection. Users found it slow and complicated to tap their mobile phone on the merchant's mobile phone. Transactions had to be reinitiated sometimes, due to NFC connection problems. These diminished user experience and led users to assign a low rating to the tasks.

Usability tests allowed for the gathering of important information concerning the design of mobile payment applications. In particular, we can say that the choice of words and phrases is very important to prevent errors and misleading actions. We noticed a marked improvement when we changed wording on the application screens. Users understood better what they could expect from each menu, preventing them from failing when performing a task [30].

### 6.3. Perceived Value

Participants have perceived the value of our payment solution in very diverse ways. The first group has mainly focused on the payment itself, highlighting the importance of performance, its value as a cash replacement and the advantages of not having to carry the wallet, cash and credit cards.

I usually never carry cash around, and it was interesting to use the mobile phone for payments, since I already save a lot of information on it as it is. (Lab_Cust2)

I find it very useful, I think it's a way of not having to carry the credit card or cash in your wallet. (Lab_Cust9)

It's a way of saving people's time, because it is very fast. It is environmentally friendly, which is very important, because it does not use paper. (Lab_Cust9)

Useful, as I don't like to carry money. I usually pay for everything with a credit card. If it is fast enough. (Ex_Cust2)

At the canteen maybe it took more time, but in other places, like at the bar, it takes more time [to pay with money or credit card] than what it took [with the mobile phone]. (Ex_Cust6)

The ability to use something that is already in our hands every day, and being able to buy daily things is great. Furthermore, it is easy to use and quick to learn. (Ex_Cust7)

On the contrary, another set of participants have mentioned that they would prefer to pay with cash and that, despite being helpful, they would not see the mobile payments app as necessary.

“It is helpful, but not necessary, because there are other payment methods that are satisfactory to me right now. And besides, you need to have specific devices that not everyone has or is willing to have.” (Lab_Cust1)

“I found it interesting. But I prefer to pay with cash”. (Lab_Cust8)

While some users seemed to associate some value with the possibility of replacing cash in their payments, problems with battery duration and with the non-ubiquitous nature of NFC payments at points-of-sale will create a situation where this type of value proposition is just not enough. Even if deployed on a larger scale, mobile payments will have to share the payment space with other forms of payment and, at least for a long time, with cash. Therefore, more than knowing how many people would prefer mobile payments to cash, our main concern was to find what type of unique value propositions participants associated with mobile payments. Identifying those value propositions could significantly help to frame the design of mobile payments towards early adoption scenarios rather than generic scenarios where they would be competing with cash. In our case, this additional value proposition was mainly centered on the ability to integrate tickets and discount coupons directly into the payment process. These features were largely appreciated by participants, particularly the possibility of buying tickets, carrying them on the mobile phone and validating them in order to gain access to services.

[…] More important than to not having to carry coins is the easiness of performing the whole process, because when riding the bus, it was quite boring having to buy the ticket. (Lab_Cust3)

I liked the part about having multiple services, like transports, canteen and others. (Lab_Cust7)

The improvement suggestions made by participants have also focused very much on this particular point. The integration with online services were the main suggestions received; buying the tickets online and then validating them with the mobile phone would save time and be friendlier to the environment. Another suggestion made was the possibility of having access to all information about payments and validations made, daily, weekly or monthly.

### 6.4. Being in Control

Fundamental to any payment process is that users and merchants can always feel that they are in full control of what is happening. When using NFC, the semantics and implications of the taps, in which the mobile devices are touched, are the core part of this control process.

Even though we had a very high success rate in the transactions made by participants, there were also some error situations. These failed transactions were due to a broad range of issues, the most common being some failure in the contact between mobile phones or network failure on the merchant's device. While we could expect this error rate to become even smaller, what was most striking from our evaluation situations was how people felt lost when it happened. With other more common payment technologies, both users and merchants have a more developed model of how the system works and what can be tried to circumvent the problem. Faced with a new technology about which they lacked the knowledge that results from previous experience, participants were clearly lost in error situations and unable to initiate any problem solving procedures, other than re-initiating the whole payment process from the beginning and hoping it would then work.

One of the strongest points in our solution was the flexibility provided by integrating payments with complimentary services, such as tickets and discount coupons. However, we have clearly observed that this flexibility comes at a cost in terms of the complexity of the procedures and cognitive overload for users in payment situations. What could be a simple procedure may become very complex from a cognitive perspective when multiple types of coupons, discounts, promotions and fidelity schemes become part of the payment transaction. While the overall procedures were considered simple by participants, coupon redemption and, to a lesser extent, the use of tickets were clearly the activities in which participants experienced more difficulties. While some of the problems could be connected with specific usability problems that could easily be overcome, it was also clear that there too was a problem with the overall mental model associated with these complimentary services.

Payments tasks, coupons, the fact that we could not see the coupons and use them directly, were a bit distrustful. (Lab_Cust3)

There are multiple sequences of procedures that could be followed to achieve essentially the same goals, and people do not have established practices for understanding how the system is supposed to work. The fact that certain operations, such as using tickets, required only one tap instead of two was also confusing:

There are situations where it only asks for one tap, and others that asks for two and that is a bit confusing. (Ex_Cust9)

The lack of an obvious reference and established practices means that people do not have an understanding about how the system is supposed to work:

In some tasks that I have been asked to perform, like using coupons, I feel that the icon metaphors and the images are misleading. (Lab_Cust1)

Coupons associate a free voucher to a bank, it does not feel correct. (Lab_Cust7)

### 6.5. Embodiment of the Payment System

A key property of NFC-based payments is the need to approach the payment terminal with the mobile device, which, in our prototype, was always another mobile phone. As a consequence, our deployment did not possess a POS optimized for intensive usage, something that seemed to be part of the expectations and frame of reference of the merchants in our study:

The payment receiving system, if it were equipped with a reading system like on supermarkets, it would be more advantageous and faster. (Ex_Mrch1)

**Figure 6.**
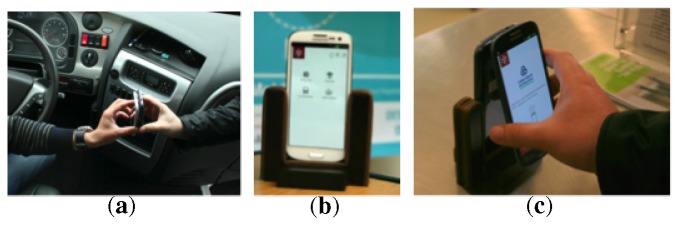
(**a**) Contact without support; (**b**) custom support; (**c**) contact with a custom support.

While we anticipate that future instantiations of the technology can have a whole different level of integration with points-of-sale, it is our understanding that one of the strong points of this payment system based on mobile phones is its ability to very quickly establish a payment system using only mobile phones. While not a relevant scenario for major stores, it may constitute a relevant value proposition for many small businesses that, due to a reduced number of sales or the inherently mobile nature of their businesses, do not justify a full POS system. In our prototype, we considered two approaches, both of which are closer to more *ad hoc* payment situations: the first was a simple and direct use of two mobile phones that were touched without any particular support for the interaction (Figure 6a); the second was a simple stand for the merchant's mobile phone (Figure 6b,c).

In the first design, both parts of the transaction had to hold their mobile phones and approach them. The main observation here is that it clearly forced participants to break the boundaries of social distance and interact at a level of proximity that was more on the range of personal or even intimate distance. The need to approach hands, align phones to achieve communication and wait in that position for the communication to occur further contributed to this feeling. While the extent to which this is a problem may vary considerably between people, it is still an issue that will always be relevant when considering this form of payment for more than occasional transactions.

In the second design, a very simple physical support was used to hold the merchant's mobile phone and make it stand in a vertical position. This would then allow the user's mobile phone to touch back-to-back with the merchant's mobile phone, while they can both check the information on their display. Our main observation here is that there was a clear trade-off in the stand design between having the merchant's mobile phone face up to facilitate interaction by the merchant or having it face down to make it easier for the user to perform the NFC communication by touching its back. The vertical position offered by our physical stand was an obvious compromise and made it not very practical for merchants to interact with the mobile phone while standing at a lower position. For this reason, merchants quickly developed the practice of holding the stand in their hand, while inserting the details of a transaction and then placing it next to the user, so that he could perform the NFC transaction. The fact that the stand was mobile gave this flexibility to the merchant, but was not so positive for users. When approaching the stand to make the NFC transaction, many people have shown some fear of pushing it away. This has negatively impacted on the performance of NFC operations, as it made the correct alignment a bit harder. With time, many users developed the practice of holding the stand while the person was approaching the mobile phone.

### 6.6. Security Perception

Participants expressed the opinion that the system is secure and that the information given by the application provides confidence and security. They indicated that asking for a PIN code in some of the transactions, receiving feedback messages, such as the message “operation successfully concluded”, receiving confirmation beeps, feeling the NFC connection vibration or even realizing the need to physically approach one mobile phone to the other, were all elements that contributed to their perception of security and trust. Having explicitly mentioned the security mechanisms integrated in our mobile payments system, users are likely to be validating those mechanisms and informing developers that initial perceptions may be mitigated in real-world contexts.

It is secure because in addition to hearing a beep, we receive a message. And then we can realize that the transactions were executed. (Lab_Cust9)

The protocol was important for the perception of security. I approached my mobile phone and received the amount to pay. I confirmed and approached the mobile phone again and received confirmation. (Lab_Cust5)

I think this is safe because it always asked for a PIN. (Ex_Cust3)

Apart from the troubles in understanding the security inherent to the payment process, almost all merchants mentioned that the fact that some of the transactions asking for a PIN gives the system a sense of security. This is curious, as merchants were never asked to enter a PIN, but they also have shown, in a real-world context, that they understand that the system must provide a sense of trust and security to users:

Because a PIN was needed, I thought it was very secure. The confirmation message is important, because otherwise we never know if the transaction was successful or not. (Ex_Mrch1)

We are always a bit distrustful at the beginning because it is a new technology and because we are dealing with money; at the beginning I was a bit confused, but with time, I realized that the system was secure. (Ex_Mrch3)

Contact with a real-word deployment of a mobile payments system was also very important for the perception that mobile payments share similar security issues with more conventional payment systems, such as debit or credit cards. Users have explicitly provided some comparisons with those systems:

In terms of safety I presume that it will be a secure system and it is like a credit card: if it got stolen, it needs a PIN, so the problems that exist in other payment methods also exist here. (Lab_Cust3)

Much more insecure than Internet payments, because it just uses a PIN. Is similar to the credit card payment. (Lab_Cust4)

## 7. Conclusions

The MobiPag pilot has demonstrated, in a real, albeit controlled environment, the main technologies and services in our mobile payments solution and has also supported, from various perspectives, the evaluation of the technology and the respective user experience.

As far as concerns technical validation, we have demonstrated how the various components that compose the MobiPag open architecture can be instantiated into a specific and complete solution for mobile payments based on NFC technology. We have built an open ecosystem that integrates a varied set of stakeholders, such as customers, merchants, mobile network operators, payment processors, banks and other technology providers. We have also validated the application model through the deployment of two reference applications for the context of the University. In addition to the inevitable integration issues, our major technical challenge was the lack of reliability of NFC. This technology is not yet stable and mature enough, leading to occasional communication failures. Even though most of the transactions have been successfully completed, those occasional ones in which an error occurred were highly frustrating for users and had a very negative influence on the overall perception of the system.

As far as concerns the user experience, the results demonstrate the broad range of elements that may have a strong influence on the overall experience and, consequently, on the value proposition of mobile payments. Considering acceptance factors regardless of these user experience elements may be useful to establish the broader value proposition of mobile payments, but it falls short in the identification of the real-world elements that may influence the perception of value. In this research, we had participants undergo a set of realistic payment situations to assess this type of element, and we have identified a number of critical design elements.

In general, we have been successful in supporting the six use cases in our trial. However, the scenario of validating a meal ticket has been revealed to be by far the most problematic. On average, all of the procedures involved in a transaction of this type were completed in 30 s. From the merchants' perspective, this was not efficient enough, as the current approach of delivering a paper ticket takes, on average, only 3 s. Moreover, the social pressure by the people standing behind in the queue was paramount. The simple prospect that something could go wrong at the moment of paying was reported by multiple users as being the only really stressful moment of the trial.

We also observed that the form factor can be a key element in these systems. This design element can often be neglected on the grounds that the payment system can be instantiated under many different form factors. While being true, the fact remains that the specific form factor being used in a deployment will constitute one of the most influential elements of the user experience with NFC and will have a major impact on the perception of the payment system.

The results in relation to the perceived value of mobile payments have confirmed the importance of doing more than just enabling payment transactions through technology. The ability to integrate tickets and discount coupons directly into the payment process were highly appreciated and clearly seen as a differentiating element that could lead to people's adoption of these systems. However, we have clearly observed that this flexibility comes with a cost in the complexity of the procedures and cognitive overload for users in payment situations. The added flexibility leading to a myriad of added services integrated with payments is simultaneously one of the strongest points and one of the biggest challenges in interaction design. What could be a simple payment procedure may become very complex for users when multiple types of coupons, discounts and loyalty schemes become part of the payment process. Further research must be done in order to analyze the impact of complex mobile service solutions on users' mental model. Furthermore, a larger deployment of an improved version of the solution should be considered in order to overcome the explorative nature of the present study.

Like other digital artifacts, mobile payment technologies should not be designed on the assumption that the frame of reference from previous payment systems can be applied directly to a new technological approach. One should acknowledge their disruptive effect on many of the practices and respective safeguards people normally resort to when making payments with currently existing methods. For example, from a technology perspective, credit cards are known to have multiple risks, but their use is based on a trust model that has evolved over the years to deal with the perception of risk by users and the management of risk by issuers. These practices have evolved and matured over the years and are now something that people trust and understand. As a new technology, mobile payments need to face the lack of knowledge about the technology itself, the lack of practices surrounding technology-based payments and the lack of well-known reference scenarios that provide confidence and trust. Another finding from our work is how important real-world usage of the technology can be in overcoming this practice vacuum and many of the potential adoption barriers. In our trial, this was particularly evident in regard to security perception. While security is clearly the number one concern in any acceptance survey on mobile payments, in our deployment, this concern was strongly mitigated by the sense that the system was actually working. Being asked for a PIN in some transactions and receiving proper feedback seems to have been enough to generate a general sense of trust in the system. Thus, other payment systems should thus focus on how to achieve a positive initial user experience and how to leverage upon real-world contact with the technology to create a solid path for gradual acceptance through the development of new practices and the perception of value.

The last general observation is the very positive attitude that participants, both customers and merchants, have shown in regard to the use of the technology. While we cannot extrapolate this into acceptance of the technology in real-world daily usage, we can assert that people are open to the new possibilities offered by mobile payments and are willing to experiment with how they can use them in realistic payment situations. The initial enthusiasm that people can demonstrate in regard to the technology means that managing expectations is crucial. First impressions should be strongly focused on quickly creating a sense of confidence, familiarity and added value with the technology that provides the foundation for sustained use and subsequent exploration of more advanced features. Designing for emerging practices and not letting people down can mean focusing at the beginning on simple procedures that, albeit limited in regards to the range of technological possibilities, are much safer and less likely to disappoint and drive users away.

## Figures and Tables

**Table 1.** Laboratory and experimental pilot: average time and average rate per task (obtained from [30]).

Tasks	Phase 1 - Laboratory Group		Phase 2 - Experimental Group	

	Avg. Time (mm: ss)	Avg. Rate	Avg. Time (mm: ss)	Avg. Rate
T1. Open MobiPag application	00:03	4.6	00:01	5.0
T2. Buy bus tickets	02:11	3.4	00:32	4.6
T3. Check bus tickets stored in the mobile phone	00:21	4.2	00:02	4.6
T4. Validate bus tickets	00:29	5.0	00:15	4.9
T5. Buy a portion of fruit	01:39	4.8	00:47	4.4
T6. Buy meal tickets	00:41	5.0	00:39	4.8
T7. Check meal tickets stored in the mobile phone	00:06	4.9	00:01	4.8
T8. Check coupons stored in the mobile phone	00:04	4.9	00:01	5.0
T9. Validate meal tickets	00:52	4.8	00:30	4.5
T10. Buy coffee (or chocolate)	01:52	2.4	00:51	4.5

## References

[1] Au Y.A., Kauffman R.J. (2008). The Economics of Mobile Payments: Understanding Stakeholder Issues for an Emerging Financial Technology Application. Electron. Commer. Rec. Appl..

[2] Kindberg T., Sellen A., Geelhoed E., Davies N., Mynatt E., Siio I. (2004). Security and Trust in Mobile Interactions: A Study of Users' Perceptions and Reasoning. UbiComp 2004: Ubiquitous Computing.

[3] Kristoffersen S., Synstad A., Sorli K. (2008). Users' perception of mobile payment. Int. J. Knowl. Manag. Stud..

[4] Mallat N., Rossi M., Tuunainen V.K., Öörni A. (2008). An Empirical Investigation of Mobile Ticketing Service Adoption in Public Transportation. Pers. Ubiquitous Comput..

[5] José R., Otero N., Rodrigues H., Meneses F., Coelho O. (2013). Exploring the Design Space of Mobile Payment Systems. Advances in Information Systems and Technologies.

[6] Chen L. (2008). A Model of ConsumerAcceptance of Mobile Payment. Int. J. Mob. Commun..

[7] Grillo A., Lentini A., Me G., Italiano G. Transaction Oriented Text Messaging with Trusted-SMS.

[8] Cassimon D., Engelen P., Yordanov V. (2011). Compound real option valuation with phase-specific volatility: A multi-phase mobile payments case study. Technovation.

[9] Mallat N. (2007). Exploring Consumer Adoption of Mobile Payments—A Qualitative Study. J. Strateg. Inf. Syst..

[10] Mallat N., Tuunainen V.K. (2008). Exploring Merchant Adoption of Mobile Payment Systems: An Empirical Study. e-Service J..

[11] Chen L., Nath R. (2004). A Framework for Mobile Business Applications. Int. J. Mob. Commun..

[12] Shin D.H. (2009). Towards an understanding of the consumer acceptance of mobile wallet. Comput. Hum. Behav..

[13] Thair A., Suhuai L., Peter S. Consumer acceptance of mobile payments: An empirical study.

[14] Kim C., Mirusmonov M., Lee I. (2010). An Empirical Examination of Factors Influencing the Intention to Use Mobile Payment. Comput. Hum. Behav..

[15] Coskun V., Ozdenizci B., Ok K. (2013). A Survey on Near Field Communication (NFC) Technology. Wirel. Pers. Commun..

[16] Zmijewska A. Evaluating wireless technologies in mobile payments—A customer centric approach.

[17] Massoth M., Bingel T. Performance of Different Mobile Payment Service Concepts Compared with a NFC-Based Solution.

[18] Ondrus J., Pigneur Y. An Assessment of NFC for Future Mobile Payment Systems.

[19] Juntunen A., Luukkainen S., Tuunainen V. Deploying NFC Technology for Mobile Ticketing Services Identification of Critical Business Model Issues.

[20] ur Rehman S., Coughlan J. (2013). An Efficient Mobile Payment System Based On NFC Technology. Int. Sci. Index.

[21] Geven A., Strassl P., Ferro B., Tscheligi M., Schwab H. Experiencing Real-world Interaction: Results from a NFC User Experience Field Trial.

[22] Chen K.Y., Chang M.L. (2013). User acceptance of ‘near field communication’ mobile phone service: an investigation based on the ‘unified theory of acceptance and use of technology’ model. Serv. Ind. J..

[23] Pasquet M., Reynaud J., Rosenberger C. Secure payment with NFC mobile phone in the SmartTouch project.

[24] Hu J.Y., Sueng C.C., Liao W.H., Ho C. Android-based mobile payment service protected by 3-factor authentication and virtual private ad hoc networking.

[25] Kadambi K.S., Li J., Karp A.H. Near-field Communication-based Secure Mobile Payment Service.

[26] Dahlberg T., Mallat N., Ondrus J., Zmijewska A. (2008). Past, Present Future of Mobile Payments Research: A Literature Review. Electron. Commer. Rec. Appl..

[27] GlobalPlatform (2012). Secure Element Access Control, version 1.0, document GPD_SPE_013.

[28] EMVCo (Europay, M.; Consortium) (2011). V. EMV 4.3 Specification. World Wide Web.

[29] Hancke G.P. (2005). A Practical Relay Attack on ISO 14443 Proximity Cards.

[30] Ferreira M.C., Cunha J.F., José R., Rodrigues H., Monteiro M.P., Ribeiro C. Evaluation of an integrated mobile payment, ticketing and couponing solution based on NFC.

